# Adapting Tree of Life Group Therapy for Vietnamese Patients with Severe Mental Disorders: A Qualitative Exploration of Treatment and Recovery Perspectives

**DOI:** 10.1192/j.eurpsy.2025.541

**Published:** 2025-08-26

**Authors:** L. T. Nguyen, H. T. T. Hoang

**Affiliations:** 1 Department of Outpatients, Hanoi Mental Hospital, Hanoi, Viet Nam

## Abstract

**Introduction:**

Severe mental disorders, characterized by their progressive course, early onset, and persistent symptoms, pose significant challenges to patients’ well-being and psychosocial functioning. Despite the growing recognition of the importance of comprehensive treatment, psychological therapies remain underutilized in this population in Vietnam. The Tree of Life therapy, a low-cost, evidence-based, and culturally adaptable intervention, offers a holistic perspective on mental health recovery through personal growth, enhanced coping skills, and social connectedness. This study aimed to explore the therapeutic potential of the Tree of Life group therapy for inpatients with severe mental disorders at Hanoi Mental Hospital.

**Objectives:**

1) To understand the experience of participating in the Tree of Life therapy group for inpatients with severe mental disorders; 2) To explore the perspectives of patients on the hospitalization, inpatient treatment process, their self-perception, life goals and resources before and after participating in the group

**Methods:**

Using qualitative methods, we interviewed 30 inpatients about their experiences before and after participating in the therapy. The Tree of Life group was conducted through four 1-hour sessions guided by the original protocol (Ncube, 2006). The study design follows a qualitative approach. After collection, the data was transcribed, coded, and stored as online text. We chose thematic analysis using MAXQDA 24 software for data analysis.

**Results:**

Regarding the Tree of Life group therapy experience, prominent themes emerged, including positive group interactions, relevant content, enhanced health and well-being, therapeutic engagement promotion, and memorable session components. When examining patients’ perspectives on hospitalization, treatment, recovery, self-description, hopes and dreams, and resources, a strong emphasis on family stood out. The family theme was then analysed further to identify subthemes: Family members were perceived as gatekeepers to treatment, sources of love and support, motivations for recovery, active participants and decision-makers in future life plans.

**Conclusions:**

The pilot qualitative study demonstrates the potential therapeutic efficacy of Tree of Life group therapy for patients with severe mental disorders. The findings also highlight the critical role of the family in supporting patients with severe mental disorders and underscore the importance of involving family members in the treatment process in the Vietnam context. However, further randomised controlled trials are required to establish the therapy’s effectiveness on a broader scale and provide robust evidence for clinical implementation.

**Disclosure of Interest:**

None Declared

